# Teacher Awareness and Attitudes Regarding Adolescent Risk Behaviours: a Sample of Finnish Middle and High School Teachers

**DOI:** 10.1007/s11469-016-9721-z

**Published:** 2016-11-22

**Authors:** Sari Castrén, Caroline E. Temcheff, Jeffrey Derevensky, Kim Josefsson, Hannu Alho, Anne H. Salonen

**Affiliations:** 1 Clinicum, Internal Medicine, University of Helsinki and Helsinki University Hospital, POB 63, 00014 Helsinki, Finland; 2 University of Sherbrooke, Longueuil, QC Canada; 3 McGill University, Montreal, QC Canada; 4 University of Helsinki, Helsinki, Finland

**Keywords:** Adolescents, Attitudes, Continuing education, Gambling, Preventive initiatives, Students, Teachers

## Abstract

Empirical evidence has shown that youth gamble on both regulated and unregulated games, despite legislative prohibitions. This study assesses middle and high school teachers’ awareness and attitudes regarding adolescent gambling and other potentially high-risk behaviours in Finland. A convenience sample of teachers (*N* = 157) from 13 provinces participated in the survey. The results suggest that teachers in Finland were more knowledgeable of the age limits of other adolescent high-risk behaviours than the legal age for gambling. Teachers were somewhat familiar with the behaviours and consequences associated with adolescent gambling. All other risk behaviours were perceived as being more important than gambling. Teachers’ awareness about gambling prevention material in Finnish schools was limited. Results suggest that initiatives are required to enhance teachers’ knowledge of adolescent problem gambling and its harmful short- and long-term consequences. School policies and guidelines including gambling behavior should be implemented in middle and high schools globally.

The liberalization, expansion and deregulation of gambling markets have led directly to a substantial increase in accessibility and availability of gambling opportunities. Regardless of legislative prohibition for underage gambling, adolescents gamble on both regulated and unregulated gambling media (Forrest and McHale 2012; Volberg et al. 2010; Blinn-Pike et al. 2010; Stinchfield 2011). There are several short- and longer-term consequences of problem gambling among adolescents, which can negatively affect adolescents’ overall social functioning, mental health, academic performance, and quality of life (Derevensky 2012; Shead et al. 2010; Castrén et al. 2015). Thus, adolescent gambling and problem gambling have been rightfully identified as public health concerns (Blinn-Pike et al. 2010; Messerlian et al. 2005). Despite this, adolescent gambling and problem gambling have been shown to be perceived as less problematic than other risk behaviours (e.g., drug use, violence in schools or spending too much time online) among parents, teachers and mental health professionals (Campbell et al. 2012; Derevensky et al. 2013; Sansanwal et al. 2015; Temcheff et al. 2014).

Adolescence is a sensitive period of life during which many risk behaviours, including gambling, are typically adopted (Dickson et al. 2004). However, unlike alcohol or other substance abuse, adolescent problem gambling is not as visible and therefore may be less easily detectable by parents, teachers, and even mental health professionals in schools (Derevensky et al. 2011), thus it is not often perceived as a form of adolescent risk behaviour (Campbell et al. 2011; Derevensky et al. 2013; Sansanwal et al. 2015, 2016; Temcheff et al. 2014). Nevertheless, internationally, the prevalence of adolescent problem/disordered gambling varies between 0.9 and 8.1 % (Volberg et al. 2010).

## Adolescent Gambling in Finland

Despite the legal gambling age of 18 years, which was raised from 15 to 18 years in 2010, with giving slot machines a 1-year transition period, which ended 2011, adolescents engage themselves in gambling activities (Salonen and Raisamo 2015). Availability and accessibility to gambling opportunities in Finland is relatively exceptional. Approximately 20 000 slot machines are not only available in gambling venues but in kiosks, shops, shopping centers, hotels, coffee shops, gas stations where youngsters have access into (Warpenius et al. 2012). Based on a population study (*N* = 4566) conducted in 2011, 44 % of 12 to18-year-old Finns had gambled during the past 6 months (Raisamo et al. 2013, 2015). Furthermore, a nationwide School Health Promotion Study (*N* = 101,167) conducted in 2010–2011 revealed that 62 % of the 14 to16-year-olds had gambled during the previous year (Räsänen et al. 2015). Depending on different methodological (e.g., severity of gambling problem measures, sampling, time) and geographical aspects, Finnish prevalence estimates for adolescent at-risk and problem gambling vary from 2.3 to 10.7 % (Ilkas and Aho 2006; Castrén et al. 2015; Räsänen et al. 2016a).

## Impact of Adults’ Attitudes on Adolescent Risk Behaviours

Given teachers spend a considerable amount of time with adolescents on a daily basis, they remain in a good position to identify youth for multiple risky behaviors. Teachers’ perceptions of adolescent risky behaviours has recently been surveyed in Canada (Derevensky et al. 2013) and Romania (Sansanwal et al. 2015). The results revealed that only 20 % of the Canadian teachers and 44.3 % of the Romanian teachers perceived gambling as a serious adolescent issue, and were the least concern compared to other potentially adolescent risky behaviours (e.g., drug use, violence or bullying, excessive online activities, excessive video game playing, smoking).

Previous research has revealed that parents’ positive attitudes towards gambling (Shead et al. 2011) and problem gambling behaviour (Delfabbro and Thrupp 2003; Magoon and Ingersoll 2006) result in increased adolescent gambling. Campbell and colleagues (2012) in Canada, reported that only 40 % of parents perceived adolescent gambling as a serious risk behaviour. In fact, adolescent gambling was rated as the least risky behaviour among 13 other potential risk behaviours, suggesting a general lack of concern regarding adolescent gambling. Similarly, more recent studies revealed that mental health professionals in schools also viewed problem gambling as the least serious adolescent risk behaviour (Temcheff et al. 2014; Sansanwal et al. 2015). At present, parents, teachers and professionals working closely with adolescents fail to perceive adolescent gambling as a high-risk behaviour (Ladouceur et al. 2004; Graham et al. 2011; Derevensky et al. 2013; Temcheff et al. 2014; Sansanwal et al. 2015, 2016).

Given the prevalence rates of adolescent gambling and problem gambling in Finland, information on teachers’ awareness, beliefs and attitudes concerning adolescent gambling and other risk behaviours is needed in order to develop school-based training and prevention materials.

The purpose of this study was to identify how Finnish middle and high school teachers perceive adolescent gambling issues and other risk behaviours. The four main areas under investigation were: a) teacher awareness of adolescent gambling; b) teacher beliefs and attitudes regarding adolescent gambling and other high-risk activities; c) teacher knowledge of school policies for gambling and other risk behavior and prevention; and d) teachers’ confidence in their ability to provide services and meet continuing education needs for youth with gambling problems*.* The results of this study will extend and further our understanding of international trends with respect to teachers’ overall knowledge and perceptions of adolescent risk behaviours, including gambling-related activities.

## Method

### Participants and Procedure

After obtaining approval from the Ethics Committee of the University of Helsinki, schools were contacted using advertisements via the Association of Finnish Principals in April 2014. Each school’s principal decided whether their school participated in the study. The principals in 19 middle and high schools informed teachers about the study, using an information letter (in which the rationale of the survey was explained and teachers were informed that their participation was voluntary, survey responses were anonymous and confidential, and that they could discontinue at any time with no consequences) and allocated time for the teachers to participate in the study. Surveys, and information letters were sent to the schools principals to be distributed. Teachers received written instructions asking them to complete the questionnaire, insert it in an envelope and place it in a sealed box in the teachers’ lounge. Surveys were completed during teachers’ meetings or lunch breaks in the teachers’ lounge, and it took approximately 25–30 min to complete. One reminder was sent in August 2014 and all completed questionnaires were sent to the researcher (SC). Of the 704 teachers invited to participate, 157 returned completed questionnaire packages (73.9 % women; response rate of 26 %).

Socio-demographic characteristics of the sample of teachers are presented in Table 1. The largest proportion of teachers was aged 50–59 years (30.8 %), and 73.7 % had taught a minimum of 10 years, thus representing a sample of experienced teachers.

**Table 1:** Socio-demographic characteristics of the respondents

Variable	*N*	Percentage (±95 %CI)
Gender		
Male	41	26.1 (±6.9)
Female	116	73.9 (±6.9)
Age		
26–29 years	16	10.3 (±4.8)
30–39 years	45	28.8 (±7.1)
40–49 years	44	28.2 (±7.1)
50–59 years	48	30.8 (±7.2)
≥ 60 years	3	1.9 (±2.1)
Teaching experience		
1–3 years	16	10.3 (±4.8)
4–6 years	21	13.4 (±5.3)
7–9 years	14	9.0 (±4.5)
10–15 years	30	19.2 (±6.2)
16–20 years	27	17.3 (±5.9)
21–25 years	16	16.7 (±5.8)
> 25 years	32	20.5 (±6.3)

### Measures

#### Awareness of Adolescent Gambling

Teachers were asked about their knowledge regarding the legal age for purchasing lottery tickets, purchasing alcohol, driving a car, purchasing cigarettes, casino gambling and playing slot machines with response options ranging from 16 to 21 years. Then, teachers’ understanding of both 11 to14-year-old and 15 to17-year-old adolescent gambling participation and gambling problems were verified with questions: ‘What percentage of youth participate in gambling-related activities at least once a year?’ and ‘What percentage of youth experience problems resulting from gambling-related activities at least once a year?’ Moreover, teachers were asked whether they had: 1) observed students participating in gambling activities, 2) overheard students taking about their participating in gambling activities, and 3) heard about students experiencing problems related to gambling during the past school year. The response options for each question were recoded into: 1) *no* or 2) *yes* (including response choices *once or twice, often, very often*). Characteristics associated with gambling problems were investigated with a question: ‘For each of the following characteristics (14 items), participants indicated the degree to which the behaviour was typical of a young problem gambler’. DSM-criteria were used as a reference for characteristics of gambling problems.

#### Beliefs and Attitudes Regarding Adolescent Gambling and High-Risk Behaviours

Teachers rated their perceptions of the seriousness of 14 adolescent potentially high-risk behaviors. The responses were recoded into two categories: 1) *not serious, somewhat serious, moderately serious* and 2) *serious or very serious*. Furthermore, teacher beliefs were queried regarding the likelihood of adolescent participation in five different high-risk activities leading to serious problems. The responses were recoded into: 1) not likely, somewhat likely, likely and 2) quite likely, very likely. In addition, teachers were asked to select the appropriate response for the statement of “Excessive gambling among young people is an important issue.” The responses were recorded into two categories: 1) *strongly disagree, disagree, neither agree/disagree* and 2) *agree, strongly agree*.

Consequences associated with youth gambling were examined with the question: “Please indicate your level of agreement with the following statements.” The statements related to gambling behavior, addictive and criminal behavior, work/school performance, friendship, self-esteem, spending time with friends, and regulation of gambling products (lottery and scratch cards) were listed. Response options were recoded into: 1) *strongly disagree, disagree, neither agree/disagree* and 2) *agree, strongly agree.* In addition, teachers were asked to indicate the extent to which they agreed with the statements regarding the acceptability of gambling for example, “There is nothing wrong with gambling occasionally” and “It is acceptable for teens to watch professional poker tournaments or TV shows featuring gambling” and were recorded as: 1) *strongly disagree, disagree, neither agree/disagree* and 2) *agree, strongly agree*. Teachers were also asked whose responsibility it was to prevent adolescents from gambling by listing eight potential choices.

#### Knowledge of School Policies for Gambling and Other Risk Behavior and Prevention

Respondents were asked about the existing policies and prevention measures for adolescent risk behaviours, including gambling, at the school level using the following questions: “Are you aware of any current policies in effect at your school / or in your school board that address the following adolescent problem behaviors (drug use, alcohol use, tobacco use, sex education, gambling, violent behaviours and eating disorders)?” Teachers were also asked to rate the importance of prevention of gambling problems and other risk behaviours within their school. Moreover, the teachers indicated whether their school had a gambling prevention program and whether their school library had gambling prevention resources.

#### Confidence in Ability to Provide Services and Continuing Education Needs

Teachers were asked about their personal involvement with students concerning gambling (e.g., “How frequently do you have conversations with your students about gambling?”), using a 5-point Likert-scale (1 = *never* to 5 = *regularly*), indicated the degree to which they felt confident that they would react appropriately if one of their students had a) gambling problem, b) alcohol problem and c) drug problem (response options included *not confident*, *somewhat confident*, *moderately confident*, *confident*, or *very confident*). Teachers also indicated whether they knew where to refer a student with gambling problems, and indicated what they thought an appropriate strategy when dealing with a student who exhibits problem gambling behaviors (e.g., helping the student address the issue or referring the student to a mental health professional). Respondents rated how important they felt it was that their school give attention to different prevention activities (e.g., not important, somewhat important, important. very important, or extremely important). Teachers were asked about their level of interest in receiving training about gambling prevention, substance addictions and violent behaviors during a professional days or on their personal time.

The original questionnaire was translated into Finnish and then back-translated into English in collaboration with the developers of the original questionnaire (Derevensky et al. 2013).

## Data Analysis

The data were analyzed using SPSS 22.0 software. Descriptive statistics including frequencies and percentage were used and 95 % confidence intervals (CI) were calculated. Cochran-Q tests of association, and logistic regression analyses were conducted to address questions in regards to differences teacher knowledge, beliefs, attitudes, and interest in obtaining further information and training. In all cases, gender differences were assed. No significant statistical differences were found between male and female teachers.

## Results

### Awareness of Adolescent Gambling

Overall, most teachers were aware of the minimum legal age for gambling-related activities. Teachers were most aware of the legal age for internet gambling (88.5 %) and slot machine gambling (85.4 %), and least aware of the legal age for purchasing a lottery ticket (72.6 %) and casino gambling (70.7 %). Overall, teachers were not well aware of the legal age for purchasing mild alcohol beverages (under 22 % alcohol, as defined by the Finnish legislation) but were quite familiar with regulatory practices (95.5 %), driving a car (99.4 %) and purchasing cigarettes (90.4 %). However, only 54.8 % of the teachers were aware of the legal age for purchasing strong alcohol beverages (containing over 22 % alcohol, as defined by the Finnish legislation).

Teachers estimated the prevalence of gambling participation and problem gambling rates in Finland (Table 2). In general, Finnish teachers estimated that 15–17-year-olds participated in gambling more often than 11–14-year-olds. Of all teachers surveyed, 16.8 % correctly estimated that approximately 41–60 % of 11–14-year-olds were likely to participate in some type of gambling at least once a year while 17.6 % of the teachers correctly noted that between 41 and 60 % of 15–17-year-olds had gambled during the past year. Of all the teachers, 35.3 % correctly estimated that the past-year problem gambling prevalence rates among 11–14-year-olds was 1–4 %; however, 16.8 % correctly estimated a similar prevalence rate among 15–18-year-olds.

**Table 2:** Teacher estimates of the percentage of adolescents who participate in gambling and who experience gambling problems

	Age group of adolescents	
	11–14 years % (±95 %CI)	15–17 years % (±95 %CI)
Teacher estimates of adolescents participating in gambling at least once a year		
14 % of youth	10.5 (±5.0)	3.5 (±3.0)
5–10 % of youth	25.2 (±7.1)	13.4 (±5.6)
11–20 % of youth	21.0 (±6.7)	11.3 (±5.2)
21–40 % of youth	17.5 (±6.2)	25.4 (±7.2)
41–60 % of youth	16.8 (±6.1)	17.6 (±6.3)
61–80 % of youth	8.4 (±4.5)	21.1 (±6.7)
81–100 % of youth	0.7 (±1.4)	7.7 (±4.3)
Teacher estimates of adolescents experiencing past-year gambling problems		
1–4 % of youth	35.3 (±8.0)	16.8 (±6.3)
5–10 % of youth	47.8 (±8.4)	45.3 (±8.3)
11–20 % of youth	10.3 (±5.1)	16.1 (±6.1)
21–40 % of youth	5.9 (±4.0)	17.5 (±6.4)
41–60 % of youth	0.7 (±1.4)	4.4 (±3.4)
61–80 % of youth	0	0
81–100 % of youth	0	0

*CI* Confidence Interval

Among all teachers, 21.9 % reported having observed students participating in gambling activities and 43.8 % had overheard students talking about their gambling at least once during the past year. Furthermore, 14.2 % of the teachers reported hearing about some student(s) experiencing problems related to gambling.

In terms of adolescent problem gambling, over half of the teachers (60.3 %) correctly identified that preoccupation with thoughts about gambling was typical of adolescent problem gamblers (Table 3). Furthermore, teachers perceived that borrowing money (51.7 %), spending an excessive amount of time gambling (48.0 %) and increasing the amount of money bet over time (41.7 %) were associated with adolescent problem gambling. Stealing money to fund gambling activities was correctly identified as a characteristic of adolescent problem gambling by only 17.9 % of teachers. Additionally, only 23.3 % of teachers identified that adolescent problem gamblers often try unsuccessfully to stop gambling as one of the symptoms for problem gambling while 54.7 % of the teachers answered that seeking strategies to win was related to problematic gambling.

**Table 3:** Teachers’ perception (*N* = 157) of typical characteristics of adolescents with gambling problems

	Diagnostic criteria	N	%(±95 %CI)
1. Thinks often about gambling	Preoccupation	91	60.3 (±7.8)
2. Seeks strategies to win when gambling	Illusion of control^a^	82	54.7 (±8.0)
3. Borrows money from others to reimburse gambling debts	Risked relationships	78	51.7 (±8.0)
4. Spends a lot of time gambling	Tolerance	72	48.0 (±8.0)
5. Increases the amount of money bet over time	Tolerance	63	41.7 (±7.9)
6. Tries unsuccessfully to stop gambling	Withdrawal	35	23.3 (±6.8)
7. Often speaks with others about their own gambling activities	–	29	19.5 (±6.4)
8. Steals money to support gambling activities	Illegal acts	27	17.9 (±6.1)
9. Has weak mathematical abilities	–	14	9.3 (±4.6)
10. Places illegal bets with the primary objective of defying the law	–	9	5.9 (±3.7)
11. Resembles a delinquent	–	8	5.4 (±3.6)
12. Uses violence in relationships	–	4	2.7 (±2.6)
13. Worries excessively about their own health	–	3	2.0 (±2.2)
14. Refrains from gambling after losing money to avoid increasing losses	–	1	0.7 (±1.3)

Not typical vs. typical (somewhat typical, mostly typical, typical, extremely typical). Modified diagnostic criteria (Fisher 2000: DSM-IV-MR-J screen)

^a^Langer (1975)

### Beliefs and Attitudes Regarding Adolescent Gambling and High-Risk Behavior

**Fig. 1:**
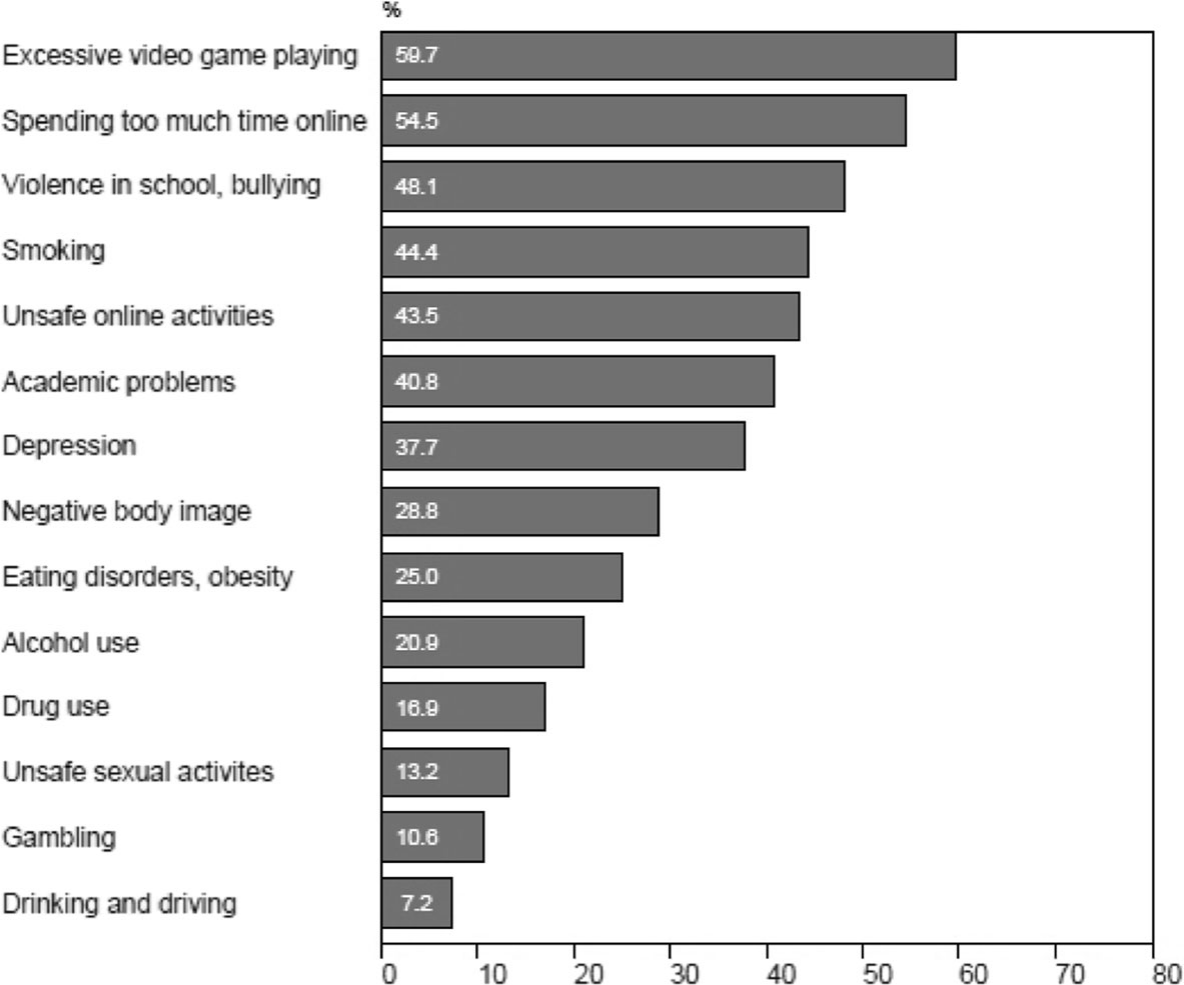
Teacher perceptions of seriousness (serious or very serious) of adolescent issues

Teachers rated the seriousness of 14 adolescent high-risk behaviours (Fig. 1). Excessive video game playing (59.7 %), spending too much time online (54.5 %) and violence in school or bullying (48.1 %) were rated as the most serious issues. Of all the potential problems, gambling was perceived as the second least serious issue (10.6 %). Despite this, 75.3 % of the teachers agreed or strongly agreed that excessive gambling remains a serious issue among adolescents.

The Cochran- Q test of association was used to detect significant differences in teacher’s perceptions of the following adolescent issues: gambling problems, drug use and abuse, alcohol use and violence in schools. There was an association between the perceived seriousness and the type of adolescent problem (*Cochran’s Q* = 98.7, *df* = 3, *p* ≤ 0.001). A logistic regression that modeled the binary response (low versus high degree of seriousness) between types of adolescent problems, using the Generalized Estimating Equations method to account for repeated measures, revealed that the odds of teacher reporting that adolescent drug use was “*serious* or *very serious*” were 1.7 times higher than reporting that adolescent gambling was either “serious tor very serious” (CI between 1.1 and 2.7 *p* = 0.02), the odds of reporting that violence in schools was “serious or very serious” was 7.8 times higher than gambling (CI between 4.6 and 13.3, *p* ≤ 0.00), and the odds of reporting that teen alcohol use was “serious or very serious” were 2.2 times higher than adolescent gambling (CI between 1.3 and 3.7, *p* ≤ 0.00).

**Fig. 2:**
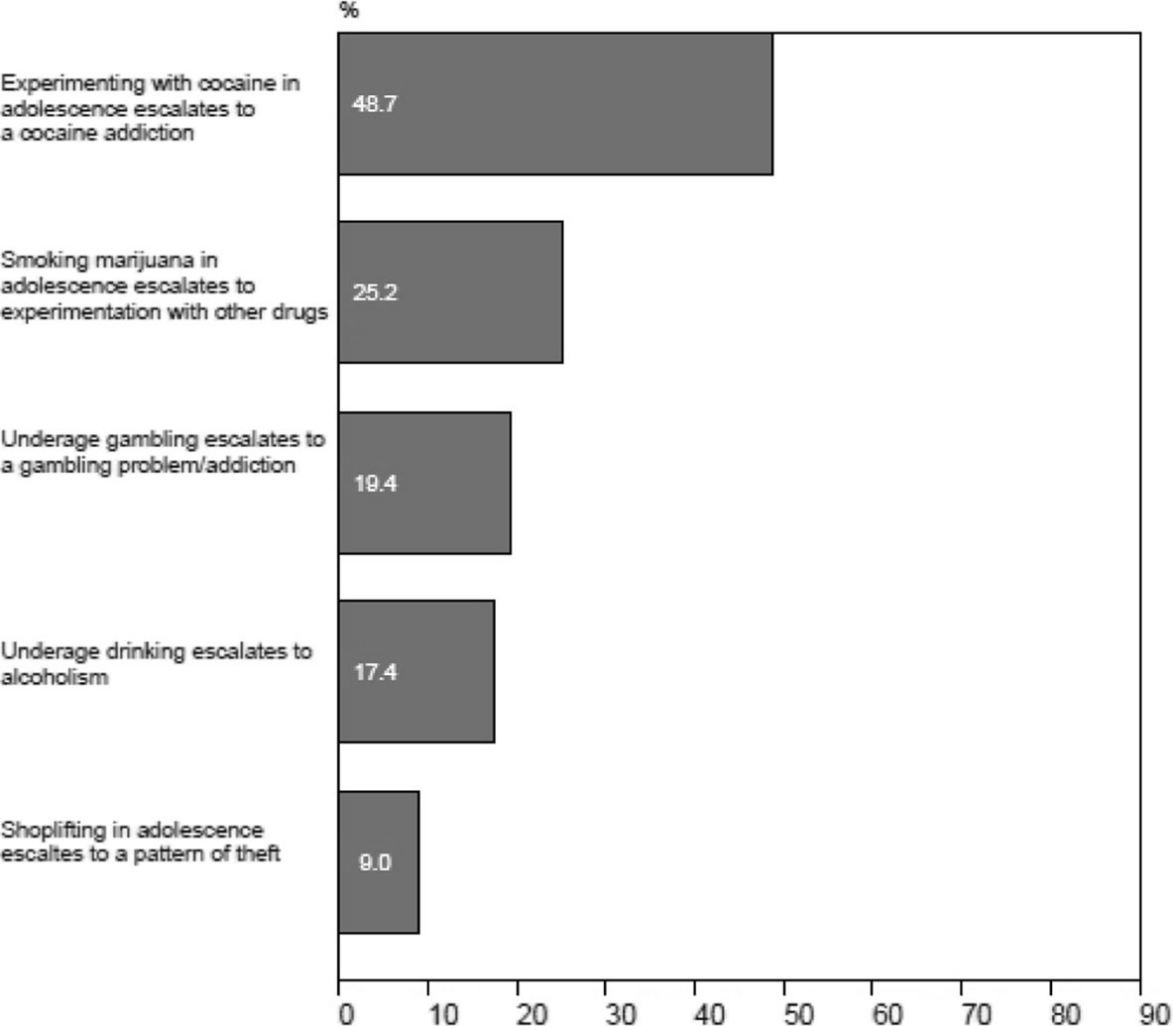
Teacher beliefs regarding the likelihood (quite likely or very likely) of adolescent participation in high-risk activities leading to serious problems

Approximately one out of five teachers (19.4 %) believed that underage gambling is likely to escalate to a problem/addiction, while almost half (48.7 %) believed that experimenting with cocaine and a quarter (25.2 %) believed smoking marijuana will lead to a problem/addiction (Fig. 2).

Teachers agreed that youth gambling can be highly addictive (84.3 %), negatively impacting both school and work performance (74.5 %) and interfering with interpersonal relationships (67.3 %). Over half of the teachers believed that gambling among adolescents could lead to criminal behaviours (53 %). Despite this finding, only 20.1 % of the teachers indicated that lottery and scratch cards in shops frequented by adolescents should be kept out of sight. Additionally, 22.2 % of the teachers viewed gambling as a possible enjoyable way to spend time with friends, 18.1 % of the teachers strongly felt that it was acceptable for teens to watch professional poker tournaments or television shows featuring gambling, and 7.8 % viewed gambling as a learning activity.

**Fig. 3:**
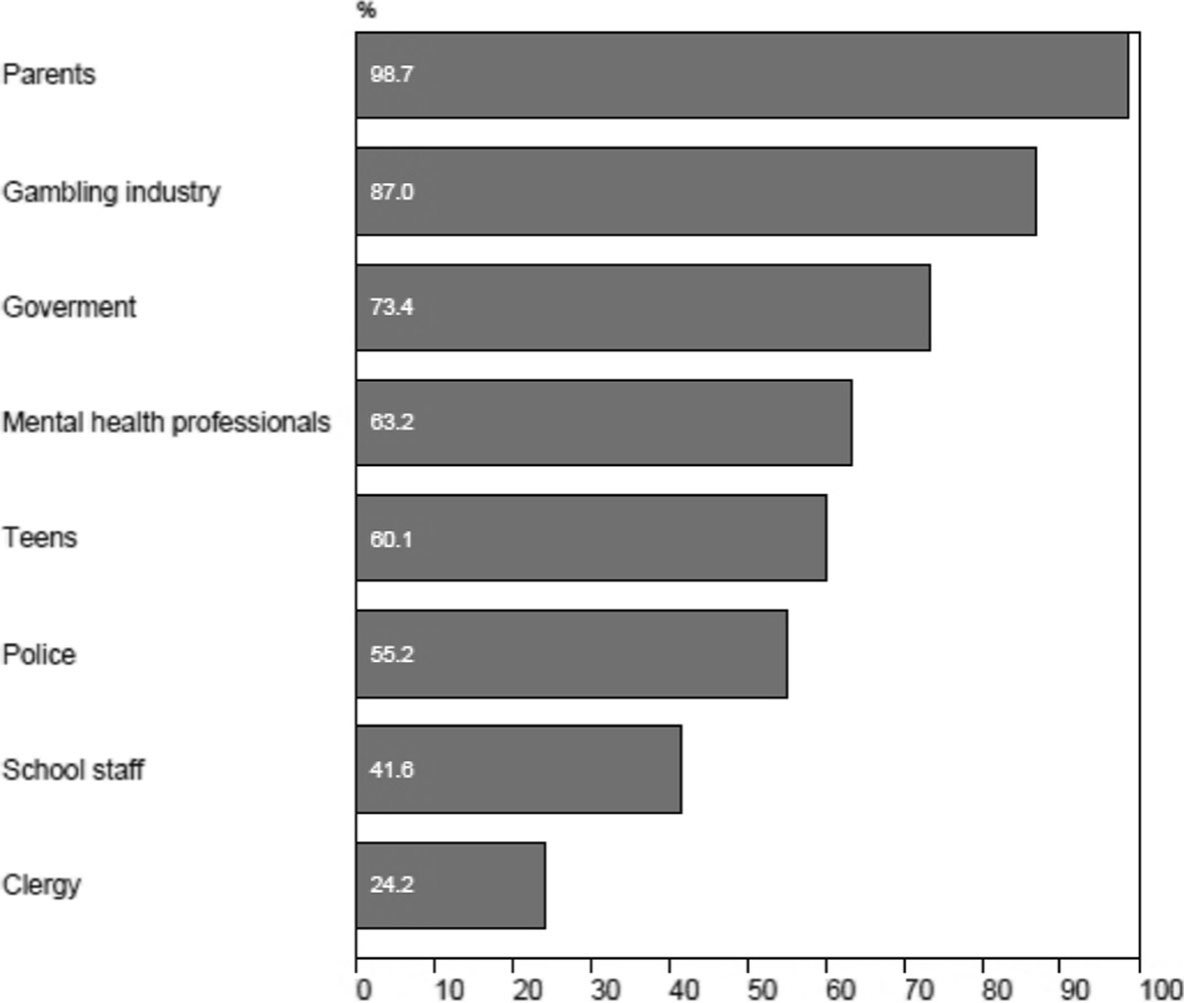
Teacher allocation of responsibility (agree or strongly agree) for the prevention of adolescent gambling

Teachers were asked whose responsibility it was to prevent adolescents from gambling (Fig. 3). The teachers perceived that parents were primarily responsible for gambling prevention (98.7 %). Most also believed that prevention was also the responsibility of the gambling industry (87.0 %) and the government (73.4 %). However, 60 % believed that the teens themselves are responsible while only 41.6 % of the teachers indicated that prevention was the responsibility of school staff.

### Knowledge of School Policies for Gambling and Other Risk Behavior and Prevention

Only 7.4 % of the teachers were aware of a gambling—related policy at their school. Furthermore, only 6.0 % of the teachers were aware of any current policies in effect in their school board that address youth gambling. Teachers were more aware of school board policies regarding violent behaviour (86.6 %), tobacco use (84.6 %) and alcohol use (72.5 %).

**Fig. 4:**
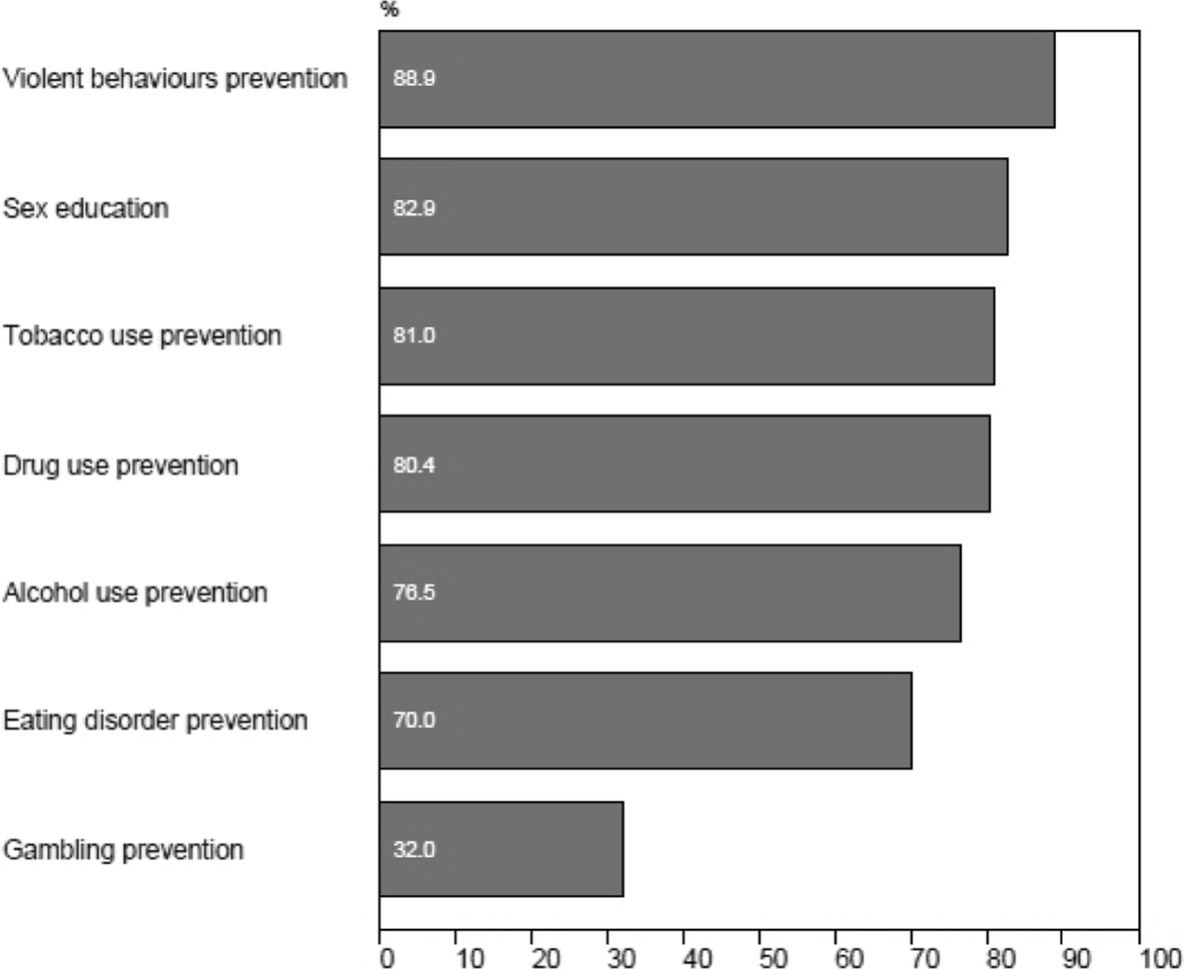
Teacher beliefs concerning the importance (very or extremely important) of the schools addressing the prevention of adolescent high-risk behaviours

In general, 32.0 % of teachers stated that it was important for the schools to address the prevention of adolescent gambling (Fig. 4) in spite of the fact that gambling prevention was considered to be the least important among all high-risk behaviours. Most teachers reported that prevention of violent behaviour in school was of significant importance (89.9 %).

None of the teachers were aware of the existence of an actual formal gambling prevention program within their school (21.4 % answered no and 74.3 % did not know). Only 5.2 % believed that their school library had any resources available for gambling prevention.

### Confidence in Ability to Provide Services and Continuing Education Needs

Among all teachers, 30.1 % reported that gambling as a topic of conversation never occurred, 48.1 % had rarely talked about it, 18.6 % sometimes talked about the issue, whereas 2.6 % of teachers often and 0.6 % regularly have had conversation about the topic. In general, 69.0 % of teachers reported feeling confident (responses *confident* or *highly confident*, henceforth called “high confidence”) in dealing with a student having a gambling problem, whereas the confidence of dealing with other addictions were 73.6 % for alcohol problem and 68.4 % for drug problems, thus there was no significant association between the type of adolescent problem and the high confidence dealing with such problems. Additionally, 57.4 % of the teachers knew of a referral resource for a student with a gambling problem (primarily the school counselor), 94.8 % reported that they would help to address this type of problem with their students. While 26.1 % would advise their students seek professional help.

**Fig. 5:**
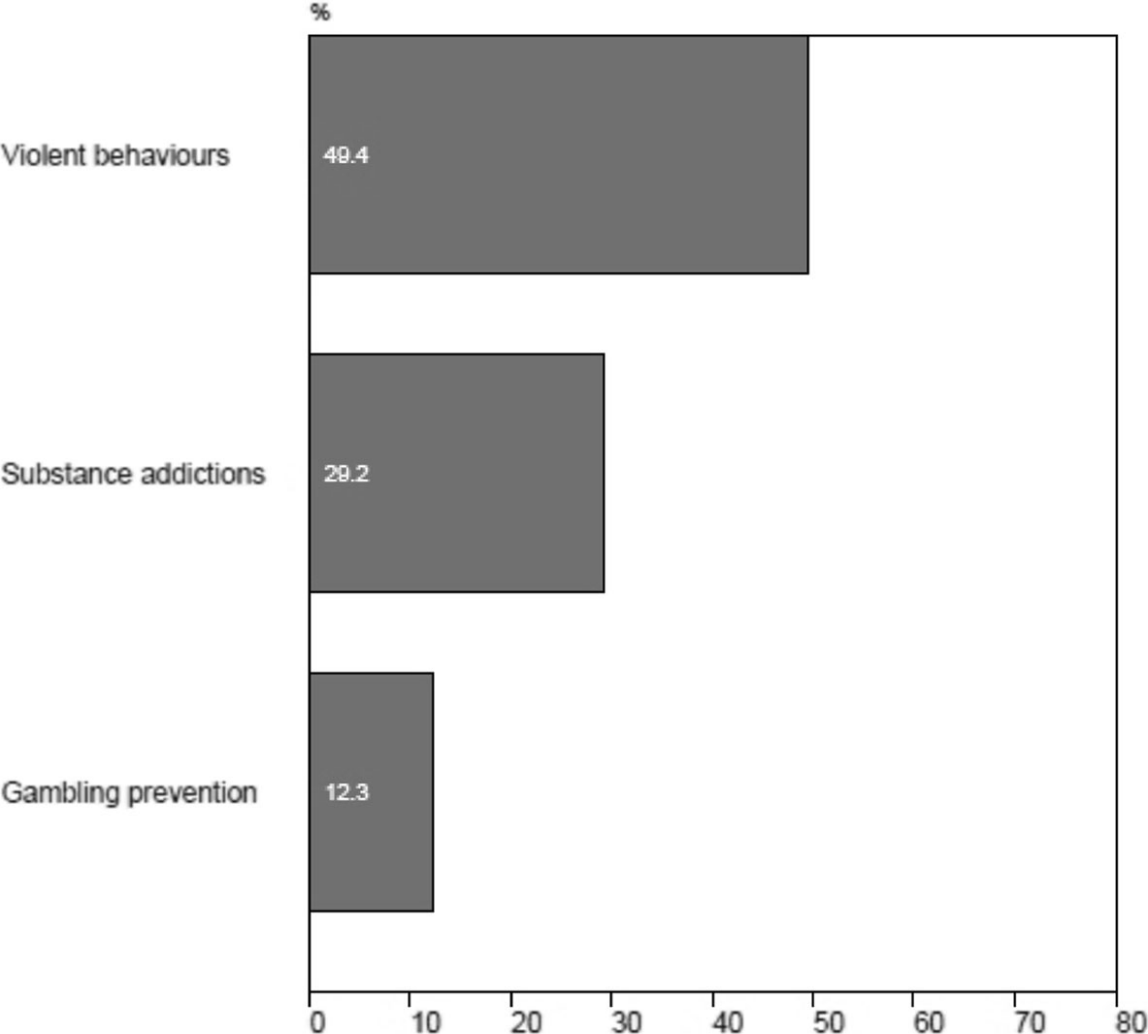
Teachers’ interest (very interested or extremely interested) in receiving training concerning adolescent high-risk behaviours during the professional day

Teachers indicated a greater interest in receiving information concerning violent behaviors in school and substance addiction than gambling prevention during professional training (Fig. 5). In order to determine whether the probability of teachers indicating being “very interested or extremely interested” (henceforth “high interest”) in receiving continuing education on gambling, substance abuse or violent behaviors during a professional day differed. There was an association between the topic of the continuing education (gambling, substance abuse or violence) and the indicated interest of teachers (*Cochran’s Q* = 81.4, *df* = 2, *p* ≤ 0.001). Furthermore, the odds ratios of reporting high interest for continuing education on substance abuse was approximately 2.9 times higher than reporting high interest for gambling (CI between 1.9 and 4.4, *p* ≤ .000), while the odds ratio of indicating high interest for continuing education for violence was 6.9 times higher than gambling (CI between 4.4 and 11.0, *p* ≤ 0.000). In addition, teachers were asked about their willingness to receive training during their personal time. Overall, their reported interest was lower, with only 1.9 % of teachers interested in receiving information about gambling, and only 13.1 % (showing interest in receiving information about violent behavior during their personal time). The probability of teachers reported “high interest” in receiving continuing education on gambling substance abuse or violent behaviors during their personal time was performed (*Cochran’s Q* = 27.7, *df* = 2, *p* ≤ 0.001). The odds of reporting high interest for continuing education on substance abuse was 2.0 times higher than gambling, but not significant (CI between .9 and 4.6, *p* = 0.08), as the odds indicating high interest for continuing education for violence was 7.6 times higher than gambling (CI between 2.5 and 23.5, *p* ≤ 0.000) in their personal time.

## Discussion

### Awareness of Adolescent Gambling

This study examined a sample of teachers’ awareness, beliefs and attitudes towards adolescent problem gambling and other risk behaviors among middle and high school students in Finland. Additionally, the teachers’ knowledge of school policies for gambling and other risk behaviors, their perceptions of their abilities to effectively deal with problem gambling among students was questioned, and their continuing education needs were addressed.

In general, teachers’ knowledge regarding the legal age for gambling-related activities was reasonably good but not quite as good as it was for example regarding purchasing alcohol and tobacco. The awareness of the legal age of purchasing gambling products (e.g., scratch cards) or to gamble is not as accurate as their knowledge of other potentially risky behaviours (e.g., purchasing alcohol, cigarettes or driving a car). This result is in line with another Finnish study, which found that enforcement of age limits was more efficient when purchasing alcohol and tobacco than gambling on slot machines (Warpenius et al. 2012).

Overall, teachers tended to over-estimate the adolescent problem gambling rates. Of importance is that a recent Finnish population study indicates that the increased mandatory age limit (including all games) on has decreased adolescent gambling and problem gambling between 2011 and 2015 (Salonen and Raisamo 2015). Yet, teachers perceptions about participation rates were somewhat correct and in line with the previous population based studies, varying from between 40 to 60 % depending on age group (Raisamo et al. 2013; Räsänen et al. 2016b). Our results highlight the same inconsistencies found in both the Canadian (Derevensky et al. 2013) and Romanian studies (Sansanwal et al. 2015) in that the teachers are to some extent aware of adolescents’ involvement, in underage gambling and recognize typical characteristics of a problem gambler, but at the same time problem gambling is viewed the least serious adolescent risk behavior. Detection of gambling-related problems are difficult, since the problems do not present as clearly as these with many other addictions (i.e., alcoholism), where the signs are more visible (Derevensky et al. 2011). It may not be due to teachers being unaware of gambling being a risky behavior, but lack of knowledge of how to identify and recognize students with possible gambling problems. Therefore, it is vital that teachers are educated on how to identify students with possible gambling related problems, how they can help with prevention and how to help them or refer them further.

### Beliefs and Attitudes Regarding Adolescent Gambling and High-Risk Behaviors

Of the 14 listed potential problems and concerns, gambling was not perceived by teachers as a very serious issue. The Finnish teachers’ perceptions about the seriousness of adolescent issues were closer to the Canadian teachers’ perceptions than the Romanian teachers’ (Derevensky et al. 2013; Sansanwal et al. 2015). However, Finnish teachers’ perceptions clearly reflected fewer concerns of any adolescent risk behaviour compared to the Canadian and Romanian teachers. This may likely reflect cross-cultural differences and general attitudes towards both adolescent issues and perceptions of gambling in different countries and a different view of overall responsibility (i.e., state, parents, educators). Overall, public attitudes towards gambling have been more favorable in Finland compared with Great Britain and Australia (Markham et al. 2015), and they have shifted even more favorable towards gambling between 2011 and 2015 (Salonen and Raisamo 2015). Gambling opportunities have increased internationally (Örnberg and Cisneros 2006; Williams et al. 2012) and are widely available and accessible in Finland. Games are not only available in casinos, but also in gambling venues such as kiosks, shops, shopping centers, hotels, coffee shops and gas stations, thus resulting in gambling becoming normalized and socially acceptable. Within the Finnish context, characterized by favorable attitudes and easy accessibility to gambling, enhancement of teachers’ awareness of problem gambling and possible harms related to it at a young and vulnerable age is important.

Similar to Canadian and Romanian teachers, Finnish teachers were concerned about excessive video game playing and time online (Derevensky et al. 2013, Sansanwal et al. 2015). Excessive video game playing, and more recently social casino gambling has been seen as a possible precursor to gambling and problem gambling. The internet provides new gambling opportunities and may become an attractive environment for adolescent gambling (Dickson et al. 2008). Many schools have already implemented ‘safe’ internet use policies teaching children about privacy issues and cyber bullying (Valcke et al. 2011). Teachers need to be provided with current information regarding the risks of gambling including internet gambling and the potential negative consequences related to these activities (Sansanwal et al. 2015).

The teachers believed the ultimate responsibility to prevent adolescents from gambling and excessive problematic gambling lies with parents, which is in-line with previous studies (Sansanwal et al. 2015, Derevensky et al. 2013). Furthermore, in Finland and Canada many teachers reported that the gambling industry had responsibility in gambling prevention for adolescents (Derevensky et al. 2013). Based on population survey among Finns aged 15 to 74, Finnish Gambling 2015, the primary responsibility to prevent gambling problems relies on individuals themselves (45.7 %), the gambling industry (32.3 %) and officials monitoring and treating gambling and gambling problems (20.3 %) (Salonen and Raisamo 2015).

### Knowledge of School Policies for Gambling and Other Risk Behavior and Prevention

Teachers were more aware of policies relating to other adolescent high-risk behaviors than those regarding gambling. Finnish teachers’ knowledge about school policies was lower compared to their international counterparts (Sansanwal et al. 2015, Derevensky et al. 2013). The awareness of formal gambling prevention program was also limited. These findings are reasonable since, currently, there are no national prevention programmes focused on adolescent gambling/problem gambling in Finland. In contrast, smoking and drinking are both well-established adolescent risk behaviours and much effort has been put into prevention programmes for tobacco smoking and alcohol abuse in Finland (Warpenius et al. 2015; Huurre et al. 2011; Raitasalo et al. 2012; Pirskanen et al. 2006; Soikkeli et al. 2015; Laine and Tossavainen 2013).

Clearly, public awareness of youth problem gambling is just emerging in Finland compared to preventive initiatives established both in Canada (i.e., International Centre for Youth Gambling Problems and High-Risk Behaviours, which provides practical tools and workshops for prevention) (St-Pierre et al. 2015; Derevensky and Gilbeau 2015; Temcheff et al. 2014; Lussier et al. 2014, Dickson et al. 2004) and programs in Romania (Lupu and Lupu 2013; Todirita and Lupu 2013; Lupu and Todirita 2013), that may in turn have been reflected in the Canadian and Romanian teachers’ perceptions of being more concerned by knowing more about the risks.

### Confidence in Ability to Provide Services and Continuing Education Needs

Notwithstanding the limited knowledge about educational resources and awareness of prevention programs for adolescent gambling, some teachers felt prepared to manage students with gambling problems. Many teachers seem to know when to refer a student to the school counselor or when to advise a student to talk to a professional. Given this, it is crucial to also address the issue with the school’s health care staff since their knowledge and awareness may be beneficial (Temcheff et al. 2014). On the other hand, a recent survey for Finnish health care and social welfare professionals indicates that their basic training does not given them enough knowledge and skills to encounter gambling problems (Castrén et al. 2016). In general, Finnish teachers’ reported relatively low interest in receiving any information relating to adolescent gambling, either in a professional training in regard to high-risk behaviours.

As suggested in earlier studies (e.g., Derevensky et al. 2013; Sansanwal et al. 2015), there is a need for school-based prevention programs addressing gambling issues similar to other high-risk behaviours such as violence and bullying issues, substance use, mental health and educational concerns including sexual education. Prevention programs for adolescents often require active participation from schools, especially in Finland, where teachers noted that there is no material available about gambling prevention or were unaware of their existence. It is important to establish policies and practical guidelines similar to those established e.g., for violent behavior, bullying and substance use in Finland. Teachers, particularly in Finland, need to be provided with current information regarding the risks of youth gambling. In Finland it would be possible to arrange in-service training times (teachers have 3 training days per year). Furthermore, strategies from the substance abuse prevention field in Finland could be used as guidelines (i.e., more efforts and initiatives, specifically aimed at schools, are needed to enhance efficacy of preventive youth work and willingness to promote students’ overall well-being) (Soikkeli et al. 2015; Vehmaskoski 2013; Laine and Tossavainen 2013).

This study is limited due to a convenience sample and relatively low participation rate. Moreover, self-reported survey methodology was used, thus the results could have been subject to participant biases. In addition, as participation was voluntary, the plausibility of self-selection biases exists. Furthermore, it must be acknowledged that cross-cultural comparison is merely descriptive since cultural aspects, such as school systems, curriculums, and teacher’s education may affect the different results in different countries.

## Conclusions and Implications

Adolescent gambling issues are not perceived as important as other well-established adolescent high-risk behaviours, despite the fact that a non-negligible percentage of Finnish adolescents suffer from gambling-related difficulties. Serious attention is needed to prevent vulnerable adolescents from gambling-related harms. Future funding and research should focus on a) increasing public awareness about youth gambling-related issues; b) offering appropriate education and training to teachers about youth gambling issues; c) adopting and implementing preventive school-based programmes; d) advocating to school boards to develop and enforce policies addressing youth gambling issues, and e) evaluating the efficacy of preventive initiatives taken.
